# A Hybrid All-Solid-State Supercapacitor Using a Dry Multilayered Graphene Oxide Electrolyte Assembly: Understanding the Charging Dynamics from Experimental and Molecular Simulation Studies

**DOI:** 10.1021/acsomega.5c00990

**Published:** 2025-10-15

**Authors:** Mawin J.M. Jimenez, Marco A.E. Maria, Leonardo M. Leidens, Alexandre F. Fonseca, Marcelo A Pereira-da-Silva, Varlei Rodrigues, Fernando Alvarez, Antonio Riul Jr

**Affiliations:** † Instituto de Física ‘Gleb Wataghin’ (IFGW), 28132 Universidade Estadual de Campinas (UNICAMP), Campinas, São Paulo 13083-970, Brazil; ‡ Federal University of São Carlos, Sorocaba, São Paulo 13565-905, Brazil; § Facens University Center, Sorocaba, São Paulo 18085-784, Brazil; ∥ Instituto de Física de São carlos−IFSC/USP, São Carlos, São Paulo 13560-250, Brasil

## Abstract

Research in supercapacitors is essential for driving innovation in energy storage, paving the way for a more sustainable and efficient future where technology and the environment coexist in harmony. Supercapacitors can instantly provide higher energy density than conventional capacitors and higher power density than batteries, despite limitations of low volumetric performance. Here, we show a simplified way to manufacture a hybrid all-solid-state supercapacitor operating at room temperature and dry conditions based on poly­(diallyldimethylammonium chloride) (PDDA)/graphene oxide (GO) multilayer assembly using the layer-by-layer technique. They display rapid discharge (relaxation time constant, τ_0_ down to 1 μs), high energy (up to 7 Wh/kg) and power (up to 1400 W/kg) densities, and specific capacitance (up to 12 F/g). Molecular dynamics simulations of a PDDA/GO system are performed with and without water molecules, highlighting the crucial role of chlorine in the system’s structure. The charge storage and fast discharge, LbL thickness control, and film conformability on practically any surface are attractive approaches in numerous practical applications. Besides simplifying the system, the exclusion of liquid electrolytes and the use of ultrathin films are advantageous in several applications, without compromising weight in structures.

## Graphical abstract

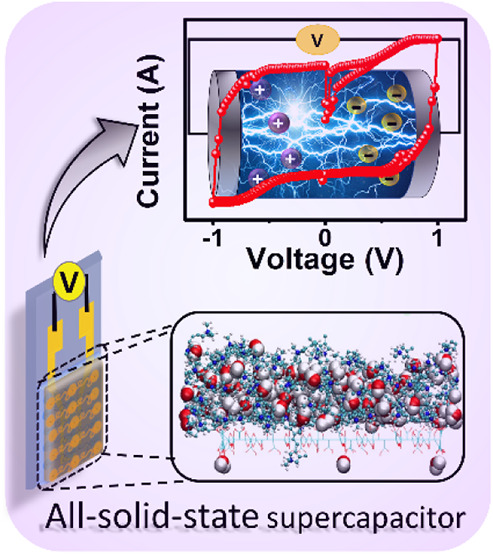

## 1 Introduction

Supercapacitors (SCs) are energy storage devices that bridge the gap between conventional capacitors and batteries. More precisely, they present higher energy densities than the former and exceed the power densities of the latter.^1,2^ The increasing proliferation of new technologies intensified the research activity in the field due to the massive market demand generated by portable devices and electric vehicles.^3,4^ Current efforts are dedicated to fabricating lighter, thinner, and faster devices with higher power and energy densities in simple configurations.^5–7^ Several processes can occur in solid-state electrodes, and depending on the charge mechanism involved, SCs can be classified as electrochemical double-layer capacitors (EDLCs), pseudocapacitors, and hybrid capacitors. In EDLCs, capacitances come from the double-layer formed through the physical adsorption of charges/ions at the electrode/electrolyte interface. The pseudocapacitive term is associated with faradaic charge transfer reactions and diffusion processes related to electronic states available at the interface.^8,9^ The hybrids combine the previous two.

At the nanoscale, both faradaic and nonfaradaic events have the same molecular origin, as the charge accumulation in the latter is not dissociated from the dynamic electron transfer in the former. Double-layer capacitors require a high surface area to favor the electrical double-layer (EDL) formation at the electrode/electrolyte interface. In pseudocapacitors, the kinetics of charge transfer and diffusion processes are related to electronic states available at the interface, i.e., accessible electronic states contributing to the charge loading of materials, modifying the electrode/electrolyte interface.^10,11^ The electrolyte’s high ionic conductivity is essential to favor charge carriers transport for superior electrochemical characteristics, but in turn, it brings electrolyte leakage, volatilization, and internal corrosion that will hamper the device performance.^12,13^ Great efforts are devoted to improving the SC design and solving liquid electrolyte problems by developing all-solid-state supercapacitors. This elegant alternative trades off lower potency for higher security (no leakage), flexibility, and miniaturization, despite utilizing more stable and environmentally friendly materials. All-solid-state supercapacitors as ultrathin films transcend consumer electronics, paving the way for the next generation of automotive vehicles and aircraft, where safety, integration, and weight reduction are absolute imperatives. The noncombustible and leak-proof nature makes them intrinsically safe to integrate into cabins and confined structures, eliminating a significant risk of catastrophic failure. More than simply replacing existing components, their revolutionary potential lies in the possibility of being formed as ultrathin films and integrated organically and invisibly into unconventional surfaces: transforming body panels, aircraft wings, sunroofs, or even interiors into active energy storage devices. This “structural electronics” capability—where a surface has energy storage functionality—allows for a drastic reduction in the weight and complexity of systems, eliminating the need for volumetric casings and connectors, paving the way for lighter vehicles (increasing electric range), more efficient aircraft and a new era of design where energy is omnipresent and invisibly embedded in the very structure of the vehicle.

In this context, numerous graphene-based materials have proven to be promising tools for energy conversion and storage devices due to their unique structures and properties.^14^ Despite the exceptional electron mobility and larger specific surface area of pristine graphene, extensive coverage is still one of the most significant challenges.^15^ Alternatively, graphene oxide (GO) has a hexagonal atomic arrangement similar to graphene but decorated with oxygenated groups (hydroxyl, epoxide, carbonyl, and carboxyl groups) covalently bonded to basal planes and edges.^16^ The oxygenated groups provide a large surface area and a hydrophilic nature that makes the material biocompatible and quickly dispersed in water.^17^ The GO production on a large scale and its easy dispersion in water favor the manufacture of low-cost supercapacitors using the Layer-by-Layer (LbL) technique. Briefly, the LbL assembly emerged as a straightforward, versatile, and inexpensive method of exploring different building blocks to form ultrathin films with excellent functionality in numerous applications.^18^ Besides, the LbL technique also enables the nanoengineering of interfaces with molecular-level thickness control, excellent homogeneity and structural organization of the films produced.^19^ The replacement of liquid or hydrogel with a dry all-solid-state electrolyte represents a commitment between optimal performance and practical operational robustness in supercapacitors. While liquid electrolytes offer superior ionic conductivity, enabling maximum power densities and low internal resistance (ESR), hydrogels represent a compromise with improved safety and flexibility. Both, however, carry inherent risks of leakage, solvent evaporation, and thermal instability. In contrast, all-solid-state supercapacitors eliminate these risks, offering unmatched safety (being nonflammable and leak-proof) and enabling the manufacture of ultrathin, flexible, and even stretchable devices. However, this advance comes at the cost of generally lower ionic conductivity of the solid electrolyte, which manifests itself as higher internal resistance and, consequently, more modest power densities and charge/discharge rates compared to their liquid counterparts. Thus, the choice between technologies boils down to prioritizing maximum performance in a rigid enclosure (liquid/hydrogel) or accepting a trade-off in performance to enable revolutionary applications in wearable, implantable, and free-form electronics, where safety and form are paramount.

Herein, we explore a gap in the literature related to ultrathin films acting simultaneously as charge accumulation material and electrolyte in the supercapacitor structure. We report a simple hybrid all-solid-state supercapacitor operating in dry conditions at room temperature, based on a multilayered poly­(diallyldimethylammonium chloride) (PDDA) and GO LbL assembly. For simplicity, the (PDDA/GO)_n_, n being the number of deposited layers, is fabricated onto gold interdigitated electrodes (IDEs). Higher capacitances are achieved in smaller areas with IDEs since metallic electrodes are at shorter distances than those arranged in parallel plate configurations. The electrical characterization performed at each deposition step during the LbL film fabrication indicated a consistent and highly reproducible hybrid all-solid-state supercapacitor. Molecular dynamic simulations of the PDDA/GO system are carried out with and without water molecules, indicating the vital role of chlorine in the system structure. Chlorine is fundamental to keeping the PDDA molecules close to GO, supporting a cohesive system. Additionally, water molecules tend to be trapped at the PDDA/GO interface, which is crucial in explaining the observed pseudocapacitive behavior. Impedance analysis of the as-fabricated SCs indicates a fast frequency response (relaxation time constant down to 1 μs), high energy density (up to 7 Wh/kg), high power density (1400 W/kg), and specific capacitance (up to 12 F/g), displaying a strong dependence on the film thickness. These characteristics, combined with thickness control and ease of deposition of LbL films on practically any surface, highlight the potential use on portable devices or even energy storage structures in vehicles without compromising weight.

## 2 Experimental Section

### 2.1 LbL Assembly

GO was chemically synthesized from Hummer’s method, and PDDA was purchased from Sigma-Aldrich and used as received. GO was dispersed in water at 0.1 g/L to form the negative polyelectrolyte, while 100 μL of PDDA was dissolved in 10 mL of water to constitute the positive polyelectrolyte. GO is negatively charged due to the presence of oxygenated groups on its structure, while PDDA is a water-soluble quaternary ammonium homopolymer having a positive charge due to ammonium (N^+^) groups. Both polyelectrolytes were prepared in ultrapure water from an Arium comfort Sartorius system (18 MΩ cm), sonicated for 20 min for complete dispersion, with their pH adjusted to 3.5 using 0.1 mol/L HCl. For the (PDDA/GO)_n_ LbL assembly substrates are first immersed 10 min in GO solution, with an intermediate washing step of 1 min in ultrapure water (also at pH 3.5) to remove weakly bonded material, followed by 10 min dipping in the PDDA solution and another washing step of 1 min in ultrapure water (also at pH 3.5). The process is repeated until the number of desired deposited layers is reached. A homemade Arduino controller does the LbL film fabrication automation, with (PDDA/GO)_n_ films easily assembled on quartz plates, gold interdigitated electrodes (IDEs), and quartz oscillators.

### 2.2 Characterizations in the LbL Assembly

All electrical characterizations were performed with LbL films deposited on IDEs photolithographically patterned onto glass slides, and fabricated at the Brazilian Nanotechnology National Laboratory (LNNano). The IDEs comprise 30 pairs of digits having 150 nm height (∼140 nm of Au onto ∼10 nm Cr adhesive layer), 3 mm length, 40 μm width, and separated by 40 μm from each other (cell constant ∼ 88 mm). The homemade LbL setup is also coupled to a Keithley 6487 picoammeter to perform current (I) versus applied potential (V) to monitor the multilayer formation at each deposition step, as previously reported in refs 20–22 . When the substrate leaves the polyelectrolytes, the picoammeter acquired an *I–V* curve in air between alternated depositions (Figure S2), using a 55 mV/s scan rate and five measurement cycles to certify the stability of the obtained experimental data. Complementary electrical impedance spectroscopy characterizations were made in a Solartron 1260A coupled to the 1296A dielectric interface, using a 25 mV sinusoidal signal, without DC polarization, at room temperature, in the 1 Hz–1 MHz frequency range. The *m* value reported in Equation 1 is determined by gravimetric mass measurements, with LbL films deposited on quartz oscillators used in a quartz crystal microbalance (QCM) coupled to a PGSTAT 302N potentiostat/galvanostat (Metrohm), also used for the galvanostatic charge–discharge.

### 2.3 Atomic Force Microscopy (AFM)

It was used for morphological analysis and was acquired in a BRUKER Dimension ICON equipment, with a rectangular-shaped silicon tip, 42 N/m spring constant, 330 kHz free oscillation, in the intermittent contact mode. Raman measurements are a powerful tool for GO characterization and were performed to performed in a Horiba Xplora equipment using the 532 nm excitation wavelength laser within the 1000–4000 cm^–1^ range, with spectra acquired on the gold fingers of the IDEs. UV–vis spectroscopy was used for GO’s complementary characterization and to follow the LbL film growth on quartz plates, being performed in a Biochrom Libra S60 spectrophotometer operating in the 200–800 nm range, with LbL films deposited onto quartz plates.

### 2.4 Raman

Raman spectroscopy was performed using a 532 nm excitation wavelength laser within the 1000–4000 cm^–1^ range in a Micro Raman Xplora Horiba. The materials were drop-casted on the IDEs, with the Raman spectra recorded on the gold fingers, guaranteeing good signal acquisition and also avoiding the strong fluorescence from glass.

## 3 Computational Details

MD simulations were performed on a set of 16 PDDA molecules, each having ten monomers, randomly placed and orientated around a GO nanosheet with dimensions of 48 Å × 48 Å, containing 30% of oxygen atoms distributed within three different chemical groups: carboxyl (COOH, 20%) on the edges of GO, and hydroxyl (OH, 40%) and epoxy (COC, 40%) distributed within the GO plane.^23^ One hundred 60 chlorine atoms were randomly displaced within the simulation box, in the exact number of nitrogen atoms belonging to the PDDA structure. Figure 8 shows the system without water molecules and chlorine ions to better view the polymer’s initial configuration around GO. Experiments having zero, 114, and 4000 water molecules simulated water’s effect on the equilibrium structure. The simulation box size was 50 Å × 50 Å × 160 Å, with GO fixed at z = 58 Å and the graphene basal plane set perpendicular to the *z*-axis. PDDA molecules were equally divided above and below GO, with water molecules randomly distributed within the simulation box in the initial configurations. The simulations were performed with periodic boundary conditions, allowing all molecules but the GO to move from one side to the other of the GO structure. All simulations consisted of: energy minimization with energy and force tolerances of 10^–4^ and 10^–6^ kcal/mol Å, respectively; an MD simulation at NVT ensemble using Nose-Hoover thermostat, with a time step of 0.5 fs, the damping factor of 100 fs, and considering a total simulation time of 4.0 ns divided in 2.0 ns of annealing +2.0 ns for system stabilization at 300 K. The annealing process was performed in two parts: 1.0 ns of simulation of the system at 700 K, and an additional 1.0 ns of simulation with the temperature reduced from 700 to 300 K. In all simulations, before collecting the properties, the total energy versus time was plotted to verify that the systems had indeed reached equilibrium (please refer to Supporting Information). The properties of the system were, then, taken at the last simulation time step.

### 3.1 MD Force Fields and Definition of the Atomic Structure

All atoms were explicitly considered for the GO nanosheet, while for PDDA, CH_3_ and CH_2_ groups were represented by superatoms with mass and charge specially chosen to represent them. The united atom approximation^24^ was used to model the interactions within GO and the PDDA systems, with force-field parameters obtained from the references.^25–27^ Water molecules were simulated using the rigid 3-sites Transferable Intermolecular Potential (TIP-3P) model,^28^ which is widely used in the literature for simulating polyelectrolytes in aqueous systems.^29,30^ The initial configuration of the system, as well as the definition of the water molecules’ positions, was built using Packmol.^31,32^ All MD simulations were carried out using the LAMMPS package.^33^

## 4 Results and Discussion

### 4.1 LbL Film Characterization

Figure 1a shows the UV–vis spectra of the GO suspension used in the LbL assembly. There is a maximum absorption band at ∼231 nm characteristic of the π-π* transition in C = C bonds present in sp^2^ regions, and a weak shoulder at ∼303 nm from n – π* transitions in C=O bonds existing in sp^3^ hybridized domains.^20^ Figure 1b illustrates the Raman spectra of a GO drop-cast film deposited on IDEs. The D band at ∼1342 cm^–1^ indicates the level of disorder or defect presented in GO nanoplatelets,^34^ and the G band at ∼1578 cm^–1^ corresponds to the vibrational E_2g_ mode involving sp^2^ carbon domains.^22^ The sharp 2D band at 2680 cm^–1^ is from a double resonance Raman scattering process, with single layers generally attributed at 2679 cm^–1^,^35^ and the D+G peak at ∼2920 cm^–1^ is defect-activated due to the presence of a disordered structure in GO.^36^ The four characteristic peaks D, G, 2D, and D + G are easily fitted by Lorentzian functions following Ferrari and Robertson,^37^ thus confirming the GO formation. The (PDDA/GO)_n_ multilayer can be successfully monitored at each deposition step with UV–vis spectroscopy with films deposited on quartz plates (Figure 1c). The inset in Figure 1c shows the dependence of the GO adsorption on the number of deposited bilayers, from the maximum absorbance value at 231 nm. The linear growth indicates the same amount of material transferred at each deposition step, endorsing the molecular-level thickness control in LbL films. QCM measurements (Figure 1d) corroborate that with a linear dependence between the deposited mass per unit area and the number of deposited bilayers, displaying an average value of ∼1075 ng/cm^2^ per deposited bilayer in (PDDA/GO)_n_ films.

**Figure 1:**
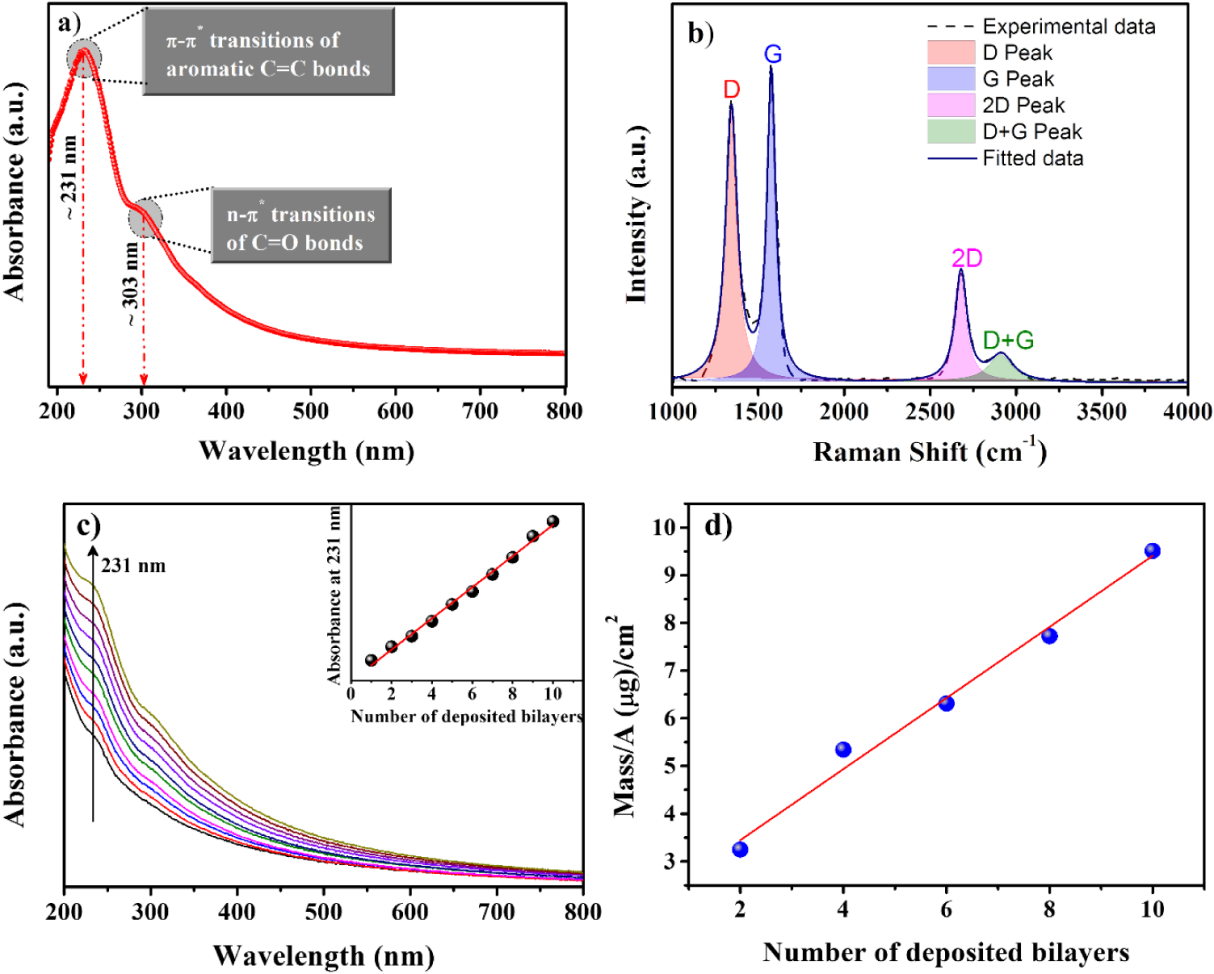
a) UV–vis absorption spectra of the GO solution and b) Raman spectra of drop-casted GO film. c) typical UV–vis absorbance spectra of layer-by-layer assembly of (PDDA/GO)_n_ deposited onto quartz substrate. Inset: evolution of the absorbance at 231 nm vs number of bilayer. d) Mass/area obtained in a quartz crystal microbalance (QCM).

AFM analysis in (PDDA/GO)_50_ LbL films (Figure 2) indicates a rough morphology and the presence of wrinkled nanoplatelets (see Figure 2a), highly keen in energy storage devices due to larger surface areas.^38^ The average RMS roughness is ∼8 nm and a total thickness of ∼230 nm (Figure 2b,c), with the film thickness measured as indicated by Faria et al.^39^ It corresponds to ∼4.6 nm per deposited bilayer, with previous studies^21^ reporting an average thickness of ∼2 nm per deposited bilayer in similar LbL architectures formed with reduced graphene oxide (rGO) and PDDA, (PDDA/rGO)_n_, keeping the same growing conditions described here. The (PDDA/rGO)_n_ structure is thinner due to the removal of oxygenated groups after the chemical conversion of GO into rGO,^40^ with an average thickness of the PDDA layer of ∼1 nm, while our PDDA/GO films retain higher hydrophilicity that enhances pseudocapacitive behavior. The oxygenated functionalities in GO also facilitate water retention, enabling hybrid charge storage mechanisms (EDL + pseudocapacitance) in the absence of a free solvent.

**Figure 2:**
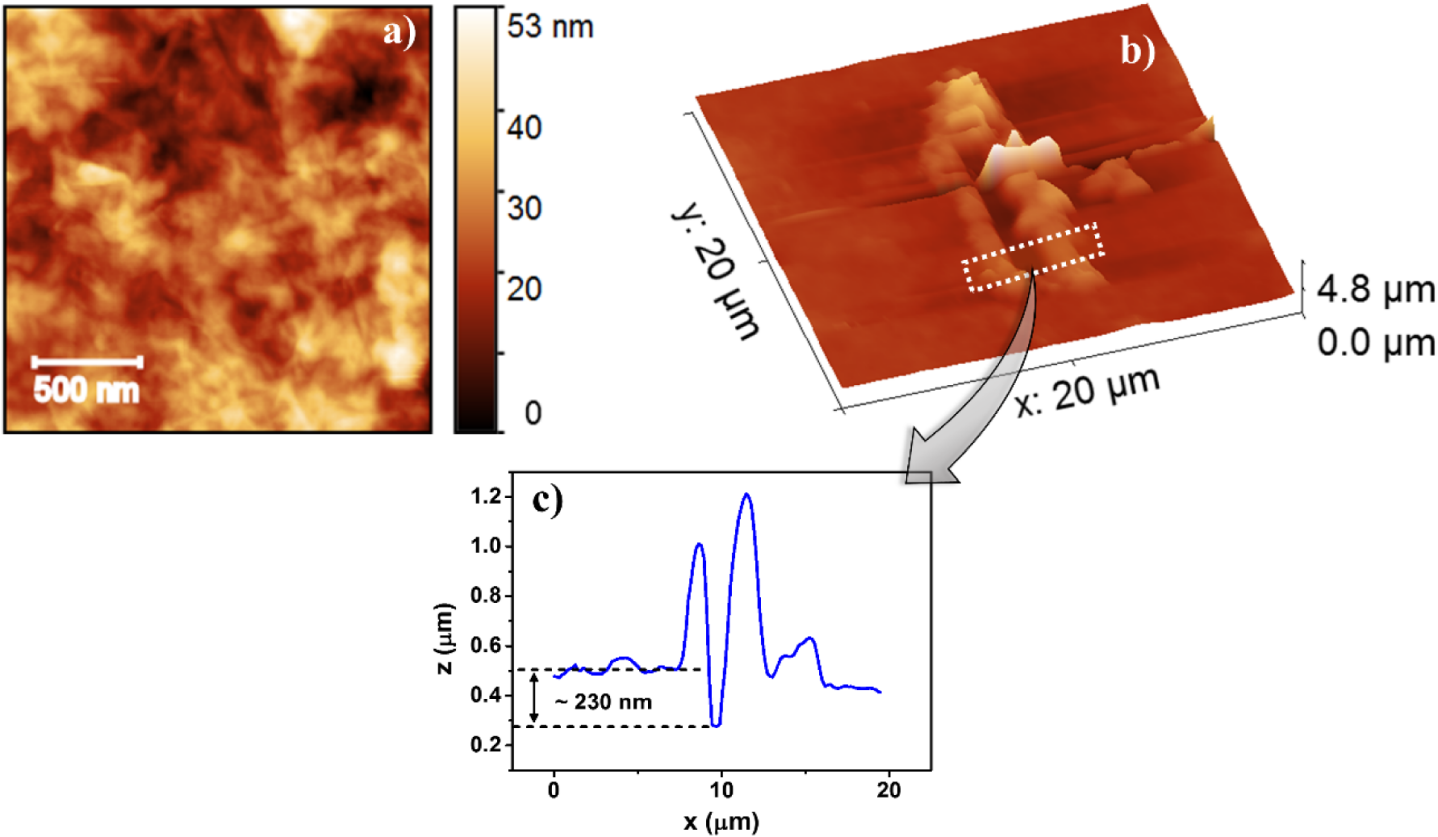
a) AFM topography; b) cutting groove with the AFM tip and c) surface profile across the groove created on a (PDDA/GO)_50_ LbL film.

The *I–V* curves at each deposition step in the LbL assembly are illustrated in Figure 3. We emphasize a significant difference in the *I–V* profile at the beginning of the LbL fabrication process related to the outermost layer. Figure 3a,b displays the first 12 deposited bilayers’ electrical responses having PDDA and GO, respectively, as the outer layer. The *I–V* plot resembles an ideal capacitor curve when PDDA is the last deposited layer, with a less pronounced, similar shape to that when GO is the outermost layer. Another important aspect is the increase in the charge accumulation capacity when PDDA is the outer layer, represented by the area under the curve.

**Figure 3:**
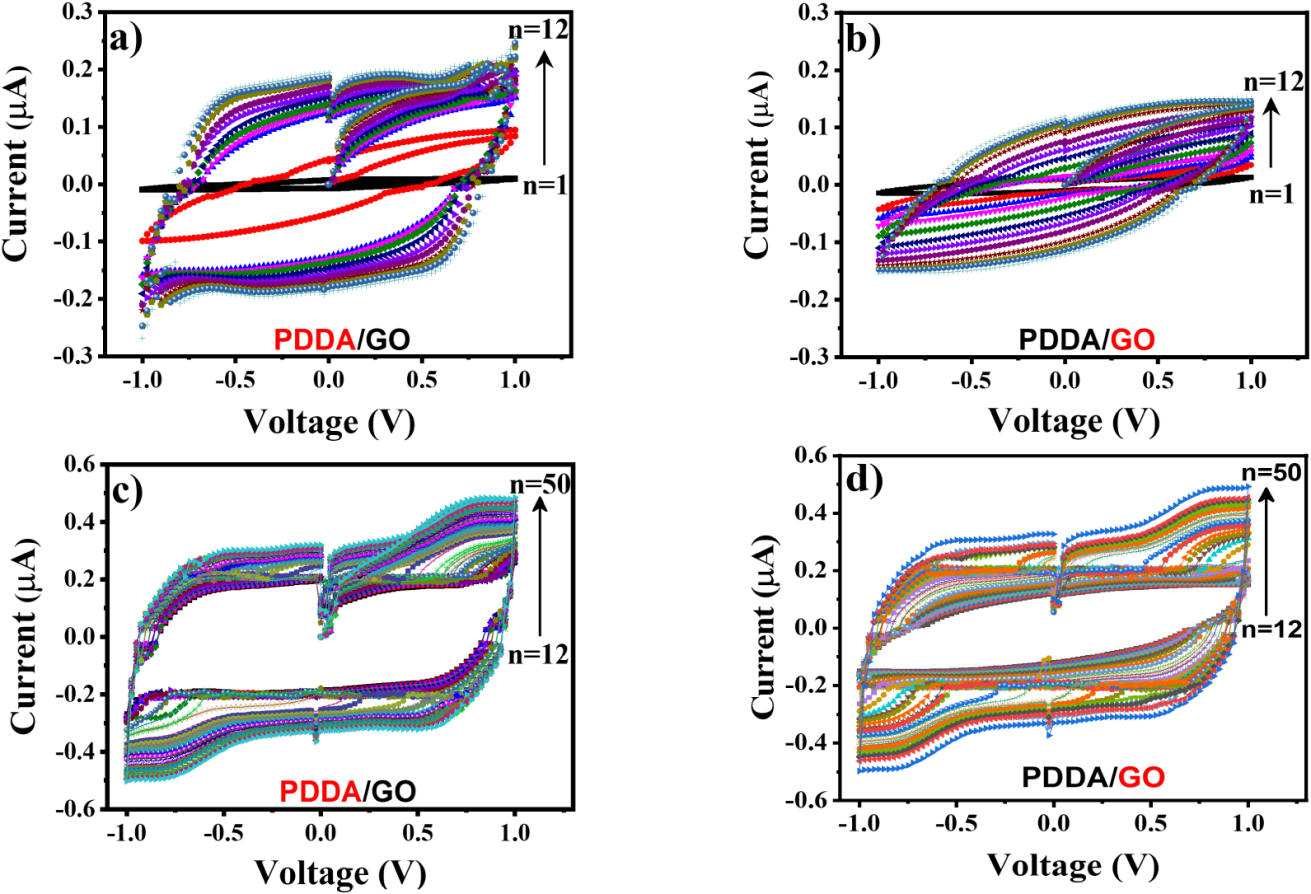
Monitoring of (PDDA/GO)_n_ LbL films with (I–V) measurements during the film assembly. Electrical behavior of the first 12 deposited bilayers: (a) PDDA as the outermost layer; (b) GO as the last deposited layer. (c) and (d) electrical response for thicker films (>12 bilayers) when we have, respectively, PDDA or GO as the outermost layer.

The pure capacitive behavior from *I–V* curves is maintained until the last deposited layer (see Figure 3c), with this format preserved even after 20 depositions, having GO as the external layer (Figure 3d). The (*I–V*) characteristics of the (PDDA/GO)_n_ LbL films are entirely reproducible, meeting the requirements of charge accumulation/depletion mechanisms in supercapacitors.^41^ Besides, electrostatic, π–π, and hydrogen bonding interactions between GO and PDDA^42,43^ make the LbL film stable enough to endure at least 500 *I–V* runs during the assembly of the multilayered structure. The deviations from ideal rectangular CV profiles at high scan rates are attributed to limited ion mobility in the dry solid matrix and interfacial polarization effects within the multilayer structure, a behavior consistent with all-solid-state devices.^44,45^ Furthermore, molecular dynamics (MD) simulations show that chlorine ions are vital to form a cohesive system structure (see Section 4.6).

### 4.2 Supercapacitor Characterization of PDDA/GO Film

The charge storage capacity, capacitance (*C, in Faraday*), specific capacitance (*C*_*s*_, *in* F/g), energy (*E, in Wh/kg*), and power densities (*P, in W/k*) of the (PDDA/GO)_n_ LbL films are determined according to^46,47^

1 $$C=\frac{1}{\nu \Delta V}\int_{V_{i}}^{V_{f}}IdV$$

2 $$C_{s}=\frac{C}{m}$$

3 $$E=\frac{1}{2}C_{s}\Delta V^{2}\left(\times \frac{1000}{3600}\right)$$

4 $$P=\frac{E}{\Delta t}\left(\times 3600\right)$$

where Δ*V*(*V*) = *V_f_* – *V_i_* is the potential window from −1 V to 1 V, ν (V/s) is the potential scan rate, $\int_{V_{i}}^{V_{f}}IdV$ is the area enclosed by the *I – V* curve, Δ*t* is the discharge time, and *m* (g) is the active mass deposited onto the IDEs.

The deduced capacitance (*C*) for each deposited layer (Equation 1) and data displayed in Figure 4a indicate an increasing capacitance with the number of deposited layers. It confirms a slightly higher capacitance when PDDA is the outer layer due to the synergistic effect of charge retention between the layers in the multilayered LbL nanostructure. Arrows indicate the same capacitance value in different situations. Briefly, (PDDA/GO)_2_, having PDDA as the outermost layer, has the same charge stored as (PDDA/GO)_11_, having GO as the last deposited layer. That happens due to screening effects inside the multilayered structure, as the polyelectrolyte charge will be screened by charge redistribution within the LbL film.^48^ Figure 4b shows the specific capacitance dependence on the number of deposited bilayers, clearly stating higher values when PDDA is the outermost layer. The overall trend is an initial rapid increase followed by an exponential decay reaching 12 F/g, then stabilizing at 3 F/g. The behavior is slightly different when GO is the outer layer, with the specific capacitance stabilized at ∼2 F/g and slightly increasing at the very end, matching the PDDA curve. Power and energy densities behave similarly to specific capacitance (see Figure 4c,d), obtaining values up to 1400 W/kg to 230 W/kg, and from 7 Wh/kg to 1 Wh/kg, respectively. Within this overall behavior, the (PDDA/GO)_2_ hybrid solid-state supercapacitor, with PDDA as the outermost layer, stands out as the highest-performing configuration, delivering a specific capacitance of 12.35 F/g, a power density of 1407.41 W/kg, and an energy density of 6.86 Wh/kg. The corresponding I–V curve is provided in Figure S3 to support these results. These parameters were calculated from the I–V data using Equation 1–Equation 4 in the main text and represent single numerical values for each deposited bilayer configuration. The inclusion of Figure S3 ensures reproducibility and substantiates the superior electrochemical performance achieved by this specific architecture. The relationship between power and energy densities of self-assembled (PDDA/GO)_n_ architectures is displayed in the Ragone plot (Figure 4e).

**Figure 4:**
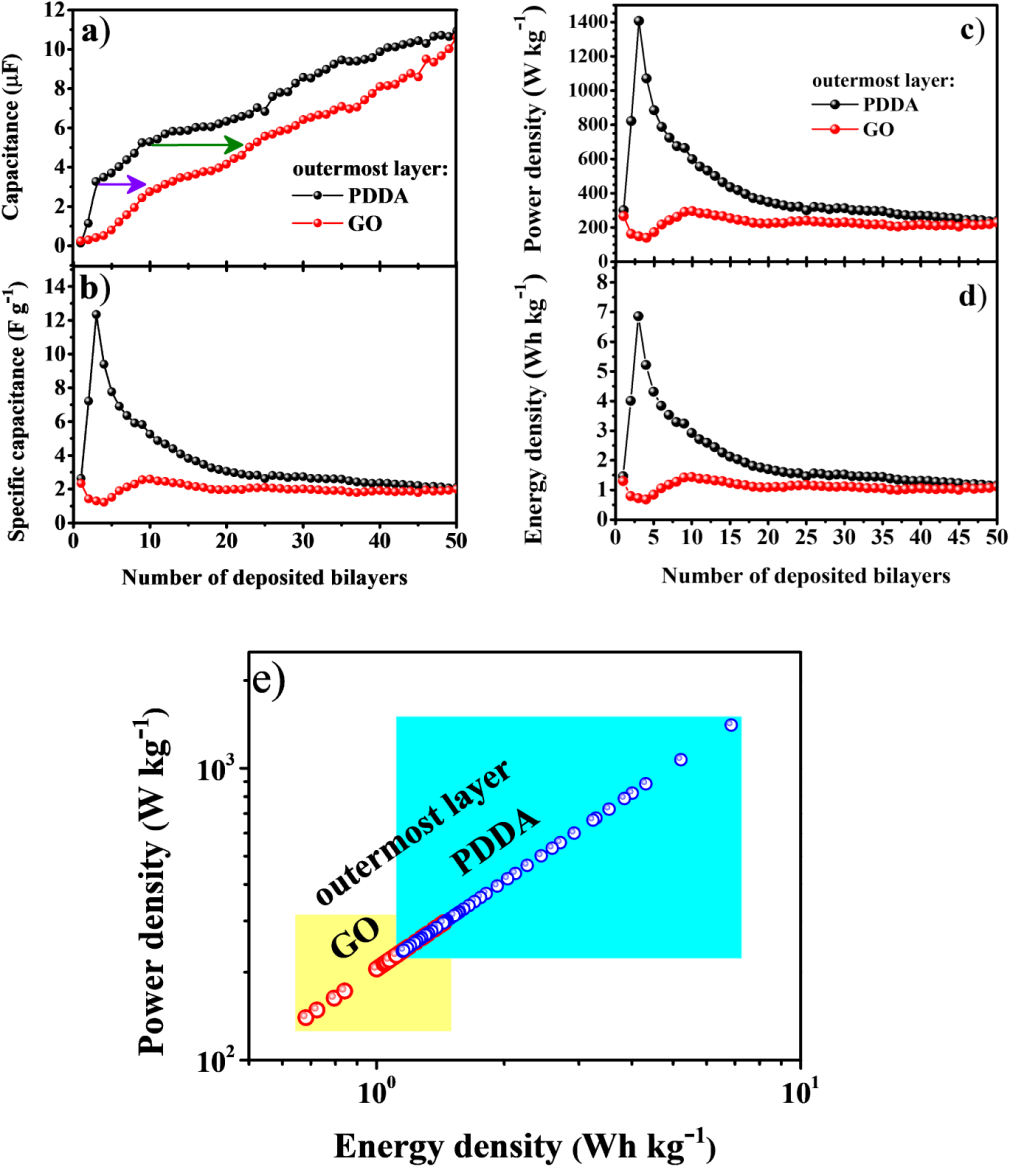
Results from the I–V characteristics of (PDDA/GO)_n_ films: a) capacitance (*C*); b) specific capacitance (*C*_*s*_); c) power density (*P*), and d) energy density (*E*). All of them are dependent on the number of deposited bilayers; e) Ragone plot for (PDDA/GO)_n_ LbL film.

The overall power and energy densities of PDDA/GO LbL film are similar to quasi-solid-state,^49^ all-solid-state,^50^ and conventional electrochemical SCs.^51^ Nowadays, the energy density value of commercial SCs is limited to ∼10 Wh kg^–1^; therefore, we still have room for optimization and adjustments depending on the thickness of the film for a specific application. It is also worth noting that the LbL films reported here are very thin, impacting the values previously reported.

The charge storage mechanism is generally attributed to the electrical double-layer formation (capacitance), faradaic reactions (pseudocapacitance), or both processes (hybrid). Cyclic voltammetry (CV) is an efficient tool for investigating the dominating kinetic processes of charge accumulation, and it is reduced here to *I – V* curves in two-contact devices. Figure 5a shows the electrical response at 10–1000 mV/s scan rates, within a −1 to 1 V potential window for (PDDA/GO)_50_. The symmetrical and nearly rectangular shape indicates capacitive behavior with rapid current response on reversal voltages. On the other hand, the specific capacitance decreased from 2.2 to 1.2 F/g as the scan rate increased from 10 to 1000 mV/s, showing its excellent performance in retaining capacitive properties at higher scanning rates. The analysis of the current dependence on the scan rate sheds some light on the charge storage kinetics. We considered the power-law relationship *I* =*aν* ^b^,^52,53^ being *I* the current at a specific sweep rate *v*, *a* constant,and *b* a critical parameter to evaluate the charge-storage kinetics. *b* = 1 indicates capacitive storage due to a double-layer process, while *b* = 0.5 is a semi-infinite diffusion-controlled faradaic process.^54–56^ Therefore, *b* can be calculated by fixing the applied potential in log *I* vs log *v* plot outcoming a straight line, with *b* being the slope of the experimental curve. The *b*-values plotted as a function of the applied potential (0.1 and 0.9 V) are in the inset of Figure 5b. The *b*-values are close to 1 at lower potentials, but approach 0.5 with increasing applied voltages. It indicates a hybrid charge storage mechanism, e.g., a charge accumulation contribution through the formation of an electrical double-layer (*I* ∼ ν), and a semi-infinite diffusion-controlled process (*I* ∼ ν^0.5^). Consequently, we run additional analyses to distinguish the current fraction originating from capacitive and pseudocapacitive mechanisms.

**Figure 5:**
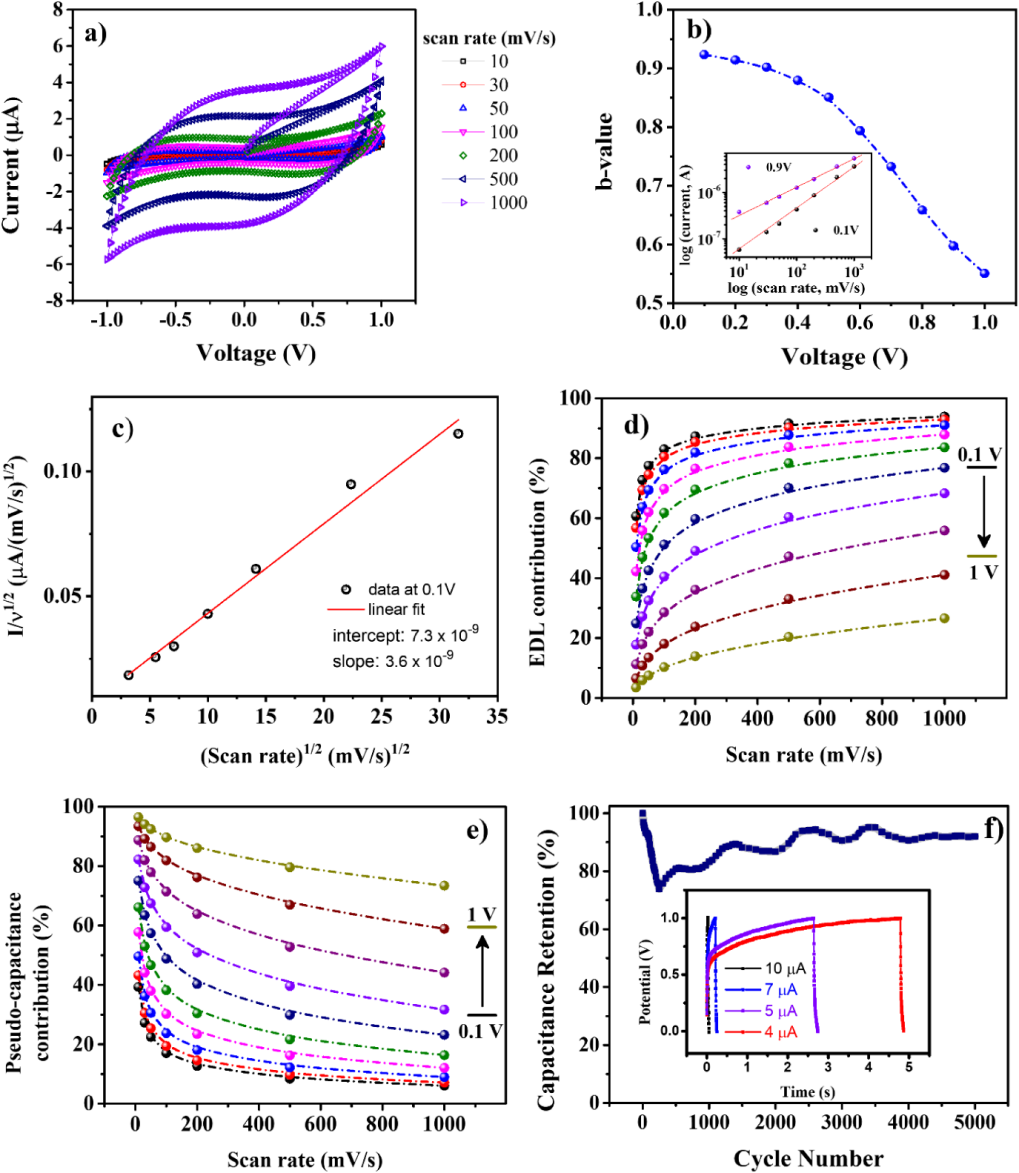
Kinetics analysis of the electrical behavior in the (PDDA/GO)_50_ hybrid solid-state supercapacitor. a) cyclic voltammograms at several scan rates; b) the calculated b-values at various potentials from 0.1 to 1 V. Inset: determination of the b-value, the current and scan rate follow the power law *I* = a*ν* ^b^. c) Linear plot of *I*/*ν* ^1/2^ as a function of square root of scan rate; d) and e) Contribution of electrical double-layer and pseudocapacitance to specific capacitance, respectively. f) Capacitance retention over 5,000 charge–discharge cycles for (PDDA/GO)_50_ film. Inset: representative galvanostatic charge–discharge curves at various current values (4–10 μA).

In this way, the response is a sum of two mechanisms contributing separately to the charge-storage kinetics according to the equation *I* = *k*_1_ *ν* + *k*_2_ *ν* ^1/2^,^57^ where *k*_1_ and *K*_2_ are constants. By obtaining both *k*_1_ *ν* and *k*_2_ *ν* ^1/2^, we can determine the contribution of capacitive and pseudocapacitive behaviors, respectively. *k*_1_ and *k*_2_ can be determined by plotting *I*/*ν* ^1/2^ vs *ν* ^1/2^ at different potentials.^58^ Figure 5c shows an example of that at 0.1 V. Figure 5d,e shows, respectively, *k*_1_ and *k*_2_ obtained in 0.1–1 V potential range, and the respective current contribution related to capacitive and pseudocapacitive behaviors calculated in each scan rate. The capacitive contribution due to the EDL formation decreases as the applied potential increases up to 1 V for all scan rates tested, with a slight increment when the scan rate increases at all potentials. An opposite behavior occurs with the pseudocapacitive contribution as it rises with increasing applied potentials and decreases slightly by increasing the scanning rate. It explains the formation of a small peak at ∼1 V in the characteristic *I – V* curves of (PDDA/GO)_n_ attributed to semi-infinite diffusion-controlled insertion processes. Figure 5f displays the stability and reliability of the (PDDA/GO)­50 solid-state supercapacitor, with an average 90% capacitance retention measured over 5,000 charge–discharge cycles. There is a decrease in the percentage of capacitance retention up to the first 250 cycles, with a gradual increase until stability is reached, as noted elsewhere.^59^ The initial drop in capacitance, followed by a gradual recovery and stabilization, may be associated with electrochemical activation and redistribution of trapped ions within solid interfaces. During early cycles, trapped species reorganize and access more favorable adsorption sites, leading to improved charge storage over time. Therefore, the increase in device capacitance is related to ion-accessible sites and ionic intercalation mechanisms in the interlayer gap, as observed in MD simulations, due to the activation progress of the materials in the LbL architecture. The ionic intercalation process is more evident with the appearance of the plateau in the charging/discharging curve for different current values (inset: Figure 5f), a characteristic behavior in energy storage devices.^60^

Moreover, as the number of deposited bilayers increases, a thicker film leads to increased ion diffusion pathways and higher resistance. This results in slower charge/discharge kinetics, which is typical in multilayered architectures. However, one advantage of this approach is the ability to tune the film thickness at the nanometer scale. This tunability is important not only for optimizing performance based on energy or power requirements but also for studying the growth kinetics of the thin film and its contribution to the overall charge carrier dynamics.

The kinetics of faradaic reactions contributing to the pseudocapacitance are not restricted to the electrode interface and occur primarily in the (PDDA/GO)_n_ film. A natural ionic intercalation process during the LbL assembly allows redox reactions by facilitating the adsorption of ions to functional groups of the materials composing the LbL structure.^61^ Besides, the accumulation of intercalated ions and electroactive species within the host bulk facilitates semi-infinite solid-state diffusion phenomena. In that sense, we perform molecular dynamic simulations of the PDDA/GO system at room temperature to get a molecular-level insight.

### 4.3 Impedance Analysis

Dielectric spectroscopy is a powerful tool to analyze a supercapacitor performance as the frequency dependency represents the charge/discharge rate due to the scanning rate of the applied potential superimposed on a low amplitude AC signal.^62^ Figure 6a illustrates the Nyquist plot of the (PDDA/GO)_50_ LbL film, with a nearly vertical straight line at the low-frequency region, characteristic of capacitive behavior. As the frequency increases, a bending point (knee frequency, *f*_*k*_) and a linear portion having a slope of ∼45° with the abscissa appear, outlining a semi-infinite diffusion at the midfrequency zone.

**Figure 6:**
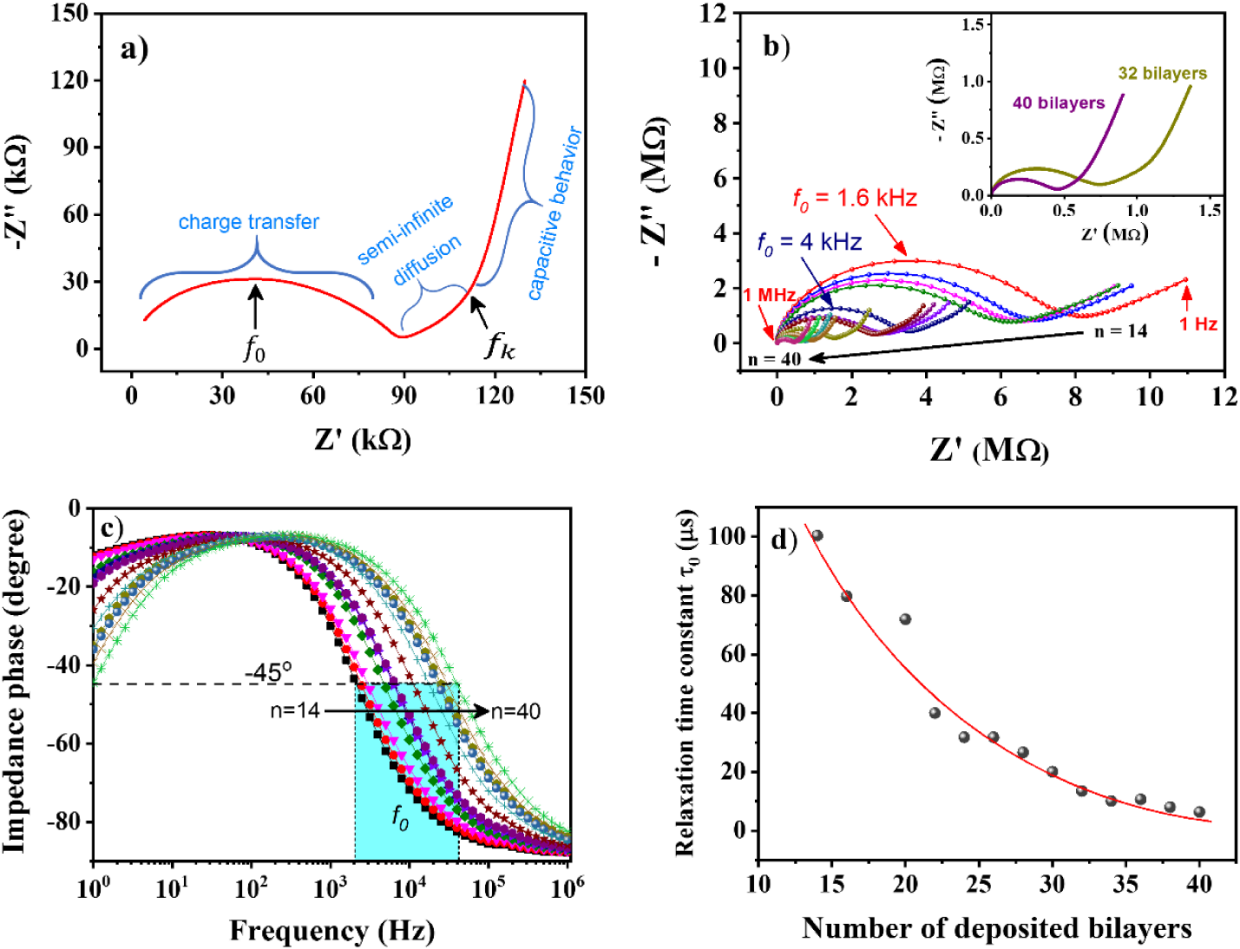
Nyquist plot of the (PDDA/GO)_50_ LbL film, b) Nyquist plots varying the number of deposited bilayers, c) Bode phase angle plots, and d) relaxation times obtained from the frequency at which the maximum peak of the Nyquist plot semicircle is formed.

This semi-infinite diffusion region is commonly attributed to the slow motion of ions and protons within solid materials,^63,64^ processes contributing to the pseudocapacitance. Then, the formation of a semicircle is characteristic of charge transfer within the LbL film structure. The maximum point in the Nyquist arch represents the relaxation frequency (*f*_0_), which sets a value for the relaxation time constant (τ_0_ = 1/2π*f*_0_). τ_0_ is an important quantitative measure of how fast the device discharges and the phase angle at −45°corresponds to a balance between real and imaginary parts of impedance. For the (PDDA/GO)_50_ film (Figure 6a), the knee point is formed at 8 Hz with characteristic frequency *f*_0_ = 145 kHz, and a constant relaxation time *τ*_0_ = 1 μs. It is a much smaller value than the 200 μs reported for a vertically oriented graphene capacitor.^65^

Moreover, the achieved charge-transfer resistance in the Nyquist plot (∼89 kΩ) allows a fast frequency response. Impedance measurements performed in (PDDA/GO)_n_ LbL film elucidates better the relationship between charge accumulation mechanisms and film thickness, as shown in Figure 6b. The vertical straight line related to the capacitive behavior at lower frequencies due to the EDL formation does not appear clearly for LbL films having less than 20 deposited bilayers. Still, it is evident the straight line associated with diffusion mechanisms that give rise to pseudocapacitance processes. As the number of deposited bilayers increases, the vertical straight line at lower frequencies appears, and the Nyquist arch tends to decrease (see inset inFigure [Fig fig6]b). Consequently, the knee point appears at higher frequencies, a behavior related to the decrease in the film resistance that allows charge transfer and diffusion of electroactive species to the electrodes’ surface. The decrease in the “radius” of the Nyquist arc means that its maximum peak is formed at frequencies higher than *f*_0_. Such *f*_0_ is best seen in the phase angle Bode plot (see Figure 6c) and appears at higher frequencies by increasing the number of deposited bilayers. Furthermore, the device at higher frequencies showed a phase angle between −88° and −82°, almost close to an ideal capacitive behavior (−90°). Accordingly, Figure 6d displays a strong dependence between τ_0_ with the number of deposited bilayers, ranging from ∼6 to 100 μs. The values reported here fit well within the supercapacitor definition range,^66^ indicating that our (PDDA/GO)_n_ hybrid all-solid-state supercapacitor can discharge rapidly with relatively high power in a dry state at room temperature. In addition, the conformability of LbL structures to practically any surface, together with the ability to adjust an all-solid-state supercapacitor’s performance with the film thickness, favors applications requiring fast recharging capacity, quite useful if applied to filling internal parts of electric vehicles or aircraft without compromising the weight of structures.

### 4.4 Equivalent Circuit Model and EIS Fitting

To gain quantitative insight into the charge transport mechanisms underlying the impedance response, the experimental EIS spectra were fitted using a transmission line equivalent circuit model adapted from Mainka et al.^67^ This approach allows us to capture the distributed ionic transport and interfacial resistances arising from the multilayered PDDA/GO assembly. The equivalent circuit, detailed in Figure 7a, consists of a series resistance (R_s_), interfacial resistances (R_i_), and two identical transmission-line cells. Each cell consists of two parallel branches between the same nodes: a constant phase element (CPE) to describe the nonideal capacitive behavior, a charge-transfer resistance R_ct_ to represents the resistance to ion rearrangement and interfacial polarization processes at the PDDA/GO interfaces, capturing the energy barrier for charge accumulation across the solid-state electrolyte layers, and a finite-length Warburg element (Z_w_) associated with restricted diffusion inside the GO nanoslits. The fitted curves reproduce the experimental Nyquist plots across all thicknesses (Figure 7b), confirming that ion transport in thin assemblies is dominated by nearly ideal capacitive behavior, whereas thicker multilayers exhibit broadened arcs and low-frequency diffusion tails. These features are consistent with the molecular dynamic simulations presented in the following section, which highlight the heterogeneous ionic mobility across confined PDDA/GO domains.

**Figure 7:**
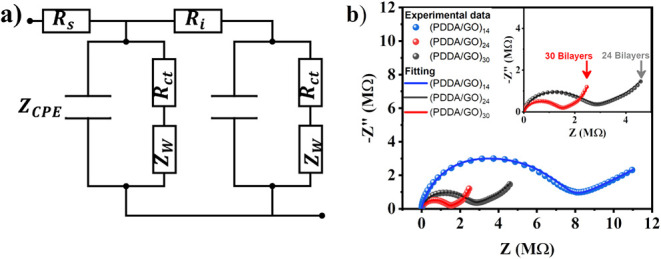
a) Equivalent transmission line circuit used for fitting the EIS data of (PDDA/GO)_n_ supercapacitors. b) Nyquist plots of experimental data and fittings for (PDDA/GO)_14_, (PDDA/GO)_24_, and (PDDA/GO)_30_. Symbols correspond to experimental data, while solid lines represent the fitting obtained using the transmission line equivalent circuit model. The inset magnifies the spectra of thicker assemblies ((PDDA/GO)_24_ and (PDDA/GO)_30_) to emphasize the increase in interfacial resistance and diffusion-related features with layer thickness.

### 4.5 Comparison of All-Solid-State (PDDA/GO)_n_ Supercapacitor with Literature Reports

To contextualize the electrochemical performance of our dry-state, all-solid-state supercapacitor, a comparative analysis was carried out against representative systems reported in the literature. Table 1 summarizes selected devices employing graphene derivatives, conducting polymers, MXenes, or carbon-based composites in combination with solid or gel-like electrolytes. The comparison includes specific capacitance, energy density, and power density, as well as the type of electrolyte and configuration. Values are presented as reported, preserving areal or volumetric units where gravimetric normalization was not provided.

**Table 1:** Comparative Performance of Representative Supercapacitors Reported in the Literature^a^

Device type	Electrode composition	Electrolyte type	Specific Capacitance	Energy Density	Power Density	ref.
Hybrid all-solid-state supercapacitor	(PDDA/GO)_n_ LbL film on gold interdigitated electrodes	Solid-State: (PDDA/GO)_n_ film	12 F/g	7 Wh/kg	1400 W/kg	This work
Flexible all-solid-state hybrid supercapacitor (FHSC)	NiCo_2_O_4_@rGO/ACF and Ti_3_C_2_T_*x*_/ACF	PVA-KOH gel	141.9 F/g	44.36 Wh/kg	985 W/kg	^68^
Asymmetric Solid-State Supercapacitor (ASSC)	N/S codoped rGO and Bi_2_O_2_CO_3_/rGO	PVA/KOH gel	68 F/g	19.85 Wh/kg	1051 W/kg	^69^
All-solid-state flexible symmetric supercapacitor	Layered MWCNTs/PANI-NTs/SWCNTs film	PVA/H_2_SO_4_ gel	258 mF/cm^2^	3.82 mWh/cm^3^	33 mW/cm^3^	^70^
All-solid-state symmetric supercapacitor (ASSSC)	rGO–PMo_12_ hybrid	0.2 M Hydroquinone (HQ) doped PVA/H_2_SO_4_ gel	4.8 F/cm^3^	17.2 Wh/kg	127 W/kg	^71^
Flexible solid-state supercapacitor	Graphene-wrapped PANI nanowire array on functionalized carbon cloth (fCC-PANI array-rGO)	PVA/H_2_SO_4_ gel	197 mF/cm^2^	0.22 mWh/cm^3^	0.50 mW/cm^3^	^72^
Solid-state flexible supercapacitor	Fe_3_O_4_/Carbon Nanotube (CNT)/PANI ternary film	PVA/H_2_SO_4_ gel	201 F/g	28.0 Wh/kg	5.3 kW/kg	^73^
Flexible solid-state supercapacitor	3D graphene-wrapped polyaniline nanofiber (rGO-PANI NF) network	H_2_SO_4_–PVA gel	211 F/g	29.3 Wh/kg	0.5 kW/kg	^74^
Flexible all-solid-state supercapacitor	Cellulose nanofibers (CNFs)/MoS_2_/RGO hybrid aerogel	H_2_SO_4_/PVA gel	657.7 F/g	22.8 Wh/kg	4.3 kW/kg	^75^
Flexible supercapacitor	Hierarchical porous graphene film (RGO) from GO hydrogel	1 M H_2_SO_4_ (liquid)	71.0 mF/cm^2^	9.8 μWh/cm^2^	∼1 mW/cm^2^	^76^
Flexible Supercapacitor	2D Hierarchical Porous Carbon Nanosheets (2D-HPCs)	EMIMBF_4_ (Ionic Liquid)	3.8 F/cm^3^	139 Wh/kg	500 W/kg	^77^
Flexible Supercapacitor	Self-supporting Activated Carbon/CNT/RGO film	1 M LiClO_4_ in EC/DEC	101 F/g	30.0 Wh/kg	∼2 kW/kg	^78^
Flexible all-solid-state supercapacitor	Cellulose Nanofibril (CNF)/RGO/CNT hybrid aerogel	H_2_SO_4_/PVA gel	252 F/g	8.1 Wh/kg	2.7 kW/kg	^79^
Transparent and Flexible Solid-State Supercapacitor	Single Walled Carbon Nanotube (SWCNT) thin films	TBAPF_6_/PMMA organic gel	34.2 F/g	18.0 Wh/kg	21.1 kW/kg	^80^
Flexible Solid-State EDLC	Activated Carbon	KOH-saturated mesoporous cellulose membrane	120.6 F/g	4.37 Wh/kg	0.25 kW/kg	^81^
Flexible All-Solid-State Supercapacitor	Carbon Nanotubes (CNTs) on RIE-treated PET	PS–PEO-PS/[EMIM][NTf_2_] ion gel	70 F/g	21.1 Wh/kg	3.0 kW/kg	^82^
Flexible All-Solid-State Micro-Supercapacitor (MSC)	N-doped reduced graphene oxide (rGO)	PVA/H_3_PO_4_ gel	3.4 mF/cm^2^	0.3 mWh/cm^3^	200 mW/cm^3^	^83^
All-Solid-State Supercapacitor	Self-assembled MWCNT film on carbon cloth	PVA/H_3_PO_4_ gel	26.8 F/g	3.5 Wh/kg	28.1 kW/kg	^84^
Flexible/Transparent Supercapacitor	Self-supporting graphene film (STF-GF) with open-hollow polyhedron units	H_2_SO_4_–PVA gel	4.21 mF/cm^2^	552.3 μWh/cm^3^	561.9 mW/cm^3^	^85^
All-solid-state symmetric supercapacitor	Reduced graphene oxide (RGO)/polypyrrole (PPy)/cellulose hybrid paper	H_3_PO_4_/PVA gel	0.51 F/cm^2^	1.18 mWh/cm^3^	∼10 mW/cm^3^	^86^
Flexible Transparent Supercapacitor	Reduced graphene oxide (rGO)/polyaniline (PANI) nanoarray nanocomposite film	H_2_SO_4_–PVA gel	4.50 mF/cm^2^	7.07 Wh/kg	707 W/kg	^87^
On-chip flexible supercapacitor	Reduced graphene oxide/polypyrrole (RGO/PPy) composite	H_2_SO_4_/PVA gel	147.9 F/cm^3^	13.15 mWh/cm^3^	1300 mW/cm^3^	^88^

a Gravimetric values (F/g, Wh/kg, W/kg) are shown when available. Areal or volumetric values are preserved where indicated to maintain consistency with the original reports . The present work is included as the first row for direct comparison .

Although several studies report higher capacitance or energy density, these values are often obtained using gel-based systems, thick and complex electrode layers, or configurations incompatible with microscale integration. In contrast, the proposed device offers full dry-state operation, microfabrication compatibility, and ultrafast charge–discharge dynamics. Its relaxation time in the microsecond range, together with the robust performance of the LbL (PDDA/GO)_n_ film architecture over interdigitated electrodes, reinforces its potential for integrated microelectronic and biosensing platforms.

### 4.6 Molecular Dynamics Simulations

We run Molecular Dynamics (MD) simulations to check the structure and dynamics of PDDA molecules around a GO nanoflake, both in the presence and absence of water molecules. The results below correspond to the PDDA molecules surrounding a GO structure after 2.0 ns of equilibrium at 300 K, considering also zero, 114, and 4000 water molecules in the simulation box, with details described in the Computational section. In all cases, GO is fixed at z = 58 Å, and the positions of the nitrogen atoms give an idea of the relative distance from the PDDA molecules to the GO nanoflake. Figure S4 shows the number of nitrogen and chlorine atoms belonging to PDDA molecules distributed along the *z*-direction (Figure 8), for the system without water molecules, which we consider a dry state. We can see that the concentration of nitrogen atoms follows the concentration trend of chlorine ions, both relatively close to GO (∼58 Å).

**Figure 8:**
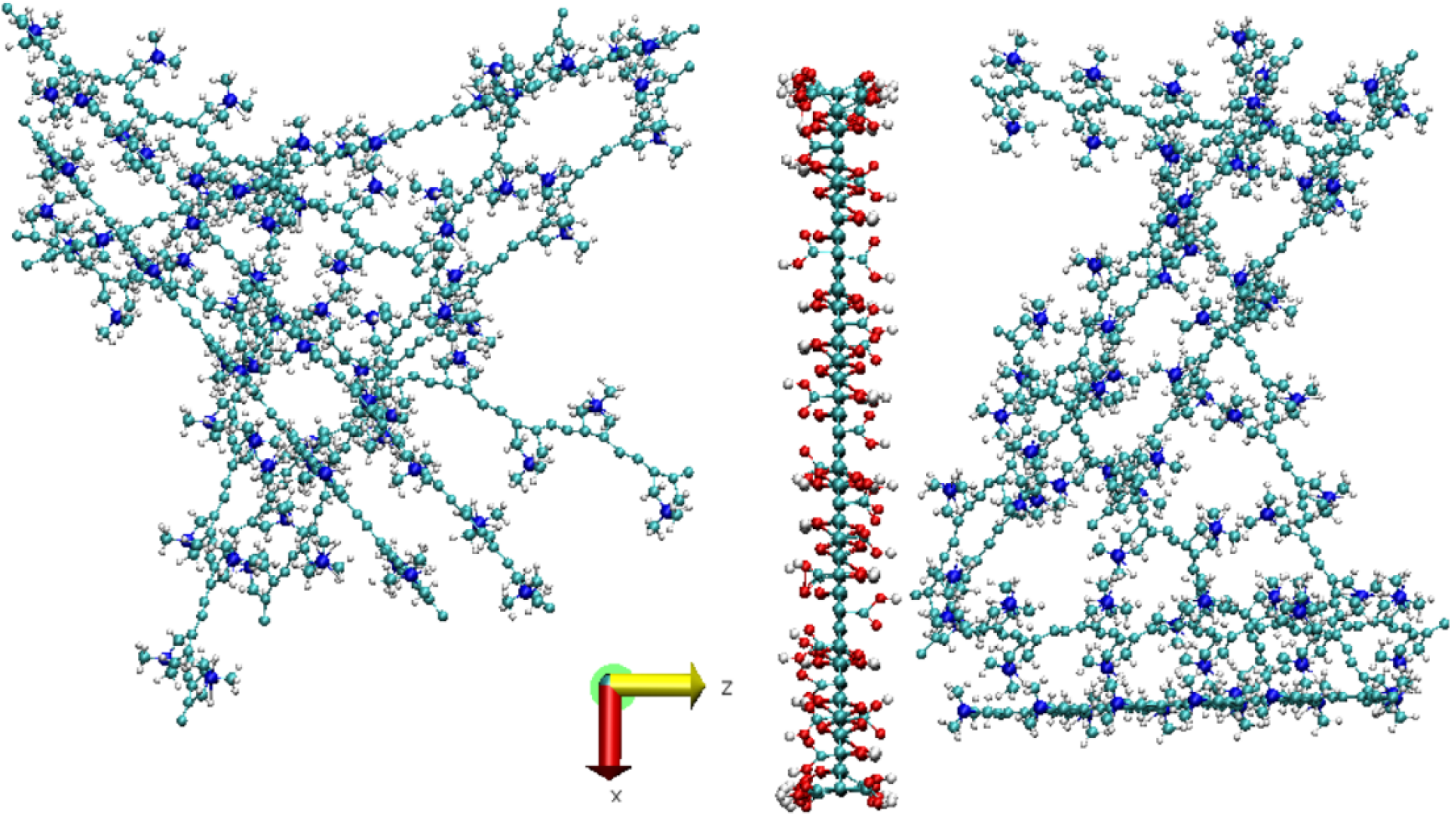
Lateral view of the initial configuration of the PDDA/GO system without water molecules and chlorine. GO is placed at z = 58 Å. Carbon, nitrogen, oxygen, and hydrogen are shown in cyan, blue, red, and white colors, respectively, and illustrated in a ball-and-stick model.

Figure S5 shows the number of chlorine and nitrogen atoms of the PDDA molecules as a function of the z coordinate within the simulation box, now considering the PDDA/GO system in equilibrium with 114 water molecules, displayed in the left panel of Figure 9. Figure S6 shows the number of chlorine and nitrogen atoms in PDDA molecules in a PDDA/GO system having 4000 water molecules within the simulation box. The inset in Figure S5 displays the number of water molecules distributed along the *z*-direction in the simulation system. Now, we see nitrogen atoms distributed slightly far from the GO nanoflake, with the PDDA molecules dispersed within the simulation box due to the increasing amount of water. Therefore, most PDDA molecules remain close to GO, despite the higher water content around the nanoflake. Once again, there is a trend of nitrogen atoms following the concentration of chlorine ions and water molecules.

**Figure 9:**
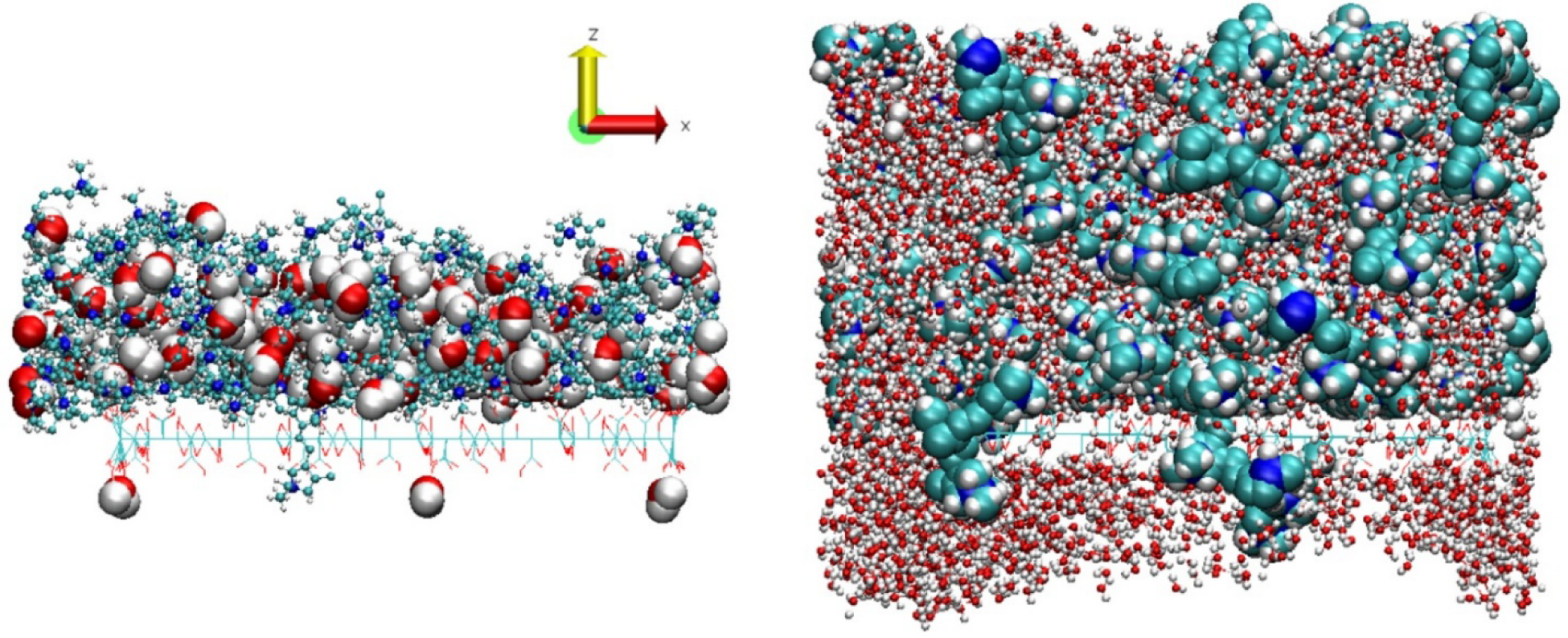
Lateral views of the equilibrated structures, at 300 K, of the PDDA/GO system with 114 (left panel) and 4000 (right panel) water molecules. GO is shown in line model in both panels. Water and polymer molecules are shown in van der Waals and balls and sticks models in the left panel, and inversely, in balls and sticks and in van der Waals models in the right panel, respectively. This inversion is to make the view of the different molecules as clear as possible given the relative number of them, one to each other. Carbon, nitrogen, oxygen and hydrogen are shown in cyan, blue, red and white colors, respectively.

The last observation of the concentration of nitrogen and chlorine atoms, which is also true for the system with a small number of water molecules, poses an important question: what attracts everything (polymer, water, and chlorine) close to GO and close to each other? Water, the chlorine ion, or GO? To answer that, we run simulations without chlorine ions. The results for the nitrogen concentration of the PDDA/GO system with 4000 water molecules, in the presence and absence of chlorine atoms, are shown in Figure S7. Without chlorine, PDDA molecules feel no significant attraction to the GO nanoflake, dispersing along the *z*-direction in the simulation box. The water molecules are concentrated around GO, but they did not pull PDDA close to the nanoflake. Therefore, we conclude that chlorine ions play a vital role in making a cohesive system, even considering a large number of water molecules. However, chlorine ions’ role in the (PDDA/GO) cohesiveness has limits. Figure S8 shows the radial distribution function, *g*(*r*), for hydrogen atoms from PDDA (Figure S8a), hydrogen atoms from GO (Figure S8b), and chlorine atoms in the system considering 114 and 4000 water molecules, respectively. Both panels a) and b) in Figure S8 show a reduction of *g*(*r*) peaks around *r* ∼ 3 Å with the addition of water molecules. It means that water molecules are screening the chlorine ions, thus reducing the attraction of both PDDA and GO to them, hampering the chlorine ion role in the system. Consequently, by increasing the water content, PDDA will spread away from GO, implying a decrease in the double-layer capacitance effect. As long as the amount of water does not become too large in the system, the water content changes the capacitance from a double-layer to a pseudocapacitive effect. An additional and indefinite increase of water into the system would ultimately extinguish the supercapacitance effects by completely dispersing the materials in the LbL structure.

The results revealed the vital role of chlorine ions in the PDDA/GO structure as they keep PDDA molecules close to GO nanoplatelets and close to themselves, considering the LbL structure assembled on that condition. The insertion of entrapped water in the PDDA/GO structure increases the dispersion around the PDDA/GO interface. It indicates that as long as the water content in the LbL structure is not massive, the double-layer capacitance decreases, with an augment in the pseudocapacitive contribution as the entrapped water content increases. The increasing water content creates a large gap between GO and PDDA, ending both faradaic and nonfaradaic effects. Lu et al.^89^ elaborated a comprehensive charge accumulation mechanism based on EDL formation in solid-state SCs. Figure S1a illustrates the initial stages of the (PDDA/GO)_n_ film formation. An applied potential (Figure S1b) starts the flow of electrons between electrodes throughout the external circuit. Initially, both electrodes have equal and opposite liquid charges, and the applied potential raises the electric field E⃗ between the electrodes. In opposition, dangling bonds, oxygenated groups, and lattice defects in GO enable the adsorption of electroactive species from water,^90,91^ and as the activation energy is close to *kT*, the adsorbed species can move along the GO basal plane.^92^ Studies indicate that water entrapped in graphene interfaces can be either by molecular or dissociative adsorption, changing the local electric field and significantly impacting the electrical properties of devices.^93^ Besides, the large GO surface area acts as an ion reservoir, facilitating the charge carrier accumulation. Under the influence of E⃗ , the electroactive species from water are attracted to interfaces with PDDA and the electrodes to form the electrical double-layer. Furthermore, PDDA is an insulator that limits transport and facilitates charge accumulation. The positively charged electrode repels positively charged groups not involved in the adsorption process with the first PDDA layer deposited onto the IDEs. It facilitates the approximation of negatively charged species from GO in the LbL structure, which in turn will facilitate the attraction of opposite charges in the subsequent PDDA layer, and so on. In other words, the PDDA matrix provides fixed quaternary ammonium groups (−N^+^(CH**_3_**)**_3_**) that strongly interact with chloride counterions, enabling electrostatic charge compensation. During the charging/discharging process, these nonsolvated Cl^–^ ions are partially mobilized within the soft PDDA matrix through segmental motion of the polymer chains, allowing dynamic reorganization near the GO interface. On the other hand, GO provides a quasi-conductive network enriched with oxygenated functional groups that facilitate limited surface redox reactions and local charge delocalization. This hybrid system thus combines electric double-layer behavior (between PDDA^+^ and Cl^–^) with mild pseudocapacitance effects at the graphene oxide (GO) surface. As previously mentioned, the process acts as a trap to water molecules at the PDDA/GO interface, and the charge accumulation in the PDDA/GO film creates an internal electric field that balances the applied electric field (E⃗) .

## 5 Conclusions

We bring a successful dry, hybrid all-solid-state SC self-assembled with a simple (PDDA/GO)­n film structure. The device displays specific capacitance up to 12 F g^–1^, rapid discharge (relaxation time constant down to 7 μs), an energy density of 7 Wh kg^–1^, and power density up to 1400 W kg^–1^. The double-layer formation explains the charge storage process at the interfaces and the synergistic effects of the multilayered PDDA/GO materials in a nanostructure. Molecular dynamic simulations showed that water molecules, with the help of chlorine ions, get concentrated preferably between GO and PDDA, which, in short, acts as a trap to water molecules in the LbL film structure. Water imprisonment is essential to maintain the semi-infinite solid-state diffusion phenomena in the reported hybrid supercapacitance, as long as its concentration is relatively low. Impedance analysis corroborates the film resistance’s dependence on the relaxation time constant and the number of deposited bilayers. The increase in the number of deposited bilayers favors the transfer of electroactive species from water and fast frequency response, indicating rapid discharge. As a final remark, we have made a simple hybrid all-solid-state supercapacitor charged with the presence of trapped water in the LbL film structure, a milestone in energy storage devices as LbL films are conformable to practically any surface without compromising structural weight in a plethora of applications.

## Supplementary Material



## Notes

Illustration of the charge storage mechanism, schematic of the (PDDA/GO)_n_ layer-by-layer assembly on IDEs and notes for molecular dynamics simulations (PDF)

## #

Facultad del Medio Ambiente y Recursos Naturales, Universidad Distrital Francisco José de Caldas, Bogotá, D.C, Colombia

## Abbreviations

GO: graphene oxide
PDDA: poly­(diallyldimethylammonium chloride)
LbL: layer-by-layer
SC: supercapacitors
EDLCs: electrochemical double-layer capacitors
EDL: electrical double-layer
IDEs: interdigitated electrodes
MD: molecular dynamic
