# Supplementary material for: A Hybrid All-Solid-State Supercapacitor Using a Dry Multilayered Graphene Oxide Electrolyte Assembly: Understanding the Charging Dynamics from Experimental and Molecular Simulation Studies
Source: ACS Omega. 2025 Oct 15;10(42):50611–25. doi: 10.1021/acsomega.5c00990 (PMC12573028; doi:10.1021/acsomega.5c00990)
Supplement: Supplementary file 1 [file ao5c00990_si_001.pdf]

Supplementary Information

Hybrid all-solid-state supercapacitor using a dry multilayered graphene oxide electrolyte assembly: Understanding the charging dynamics from experimental and molecular simulation studies

Mawin J.M. Jimenez[*,1]*, Marco A.E. Maria*[1,2,3]*, Leonardo M. Leidens*[1]*, Alexandre F. Fonseca*[1]*, Marcelo A Pereira-da-Silva*[4]*, Varlei Rodrigues*[1]*, Fernando Alvarez*[1]*, Antonio Riul Jr*[*,1]

[1]Department of Applied Physics, '*Gleb Wataghin*' Institute of Physics (IFGW), University of Campinas—UNICAMP, 13083-970, Campinas, SP, Brazil.

[2]Federal University of São Carlos – Sorocaba.

[3]Facens University Center – Sorocaba.

[4]Instituto de Física de São Carlos—IFSC/USP, 13560-250, São Carlos, SP, Brasil.

* Correspondence to: riul@unicamp.br; mjmj@ifi.unicamp.br

This Supplement contains:

Supplementary Figures S1 to S8

Supplementary Notes for Molecular Dynamics simulations

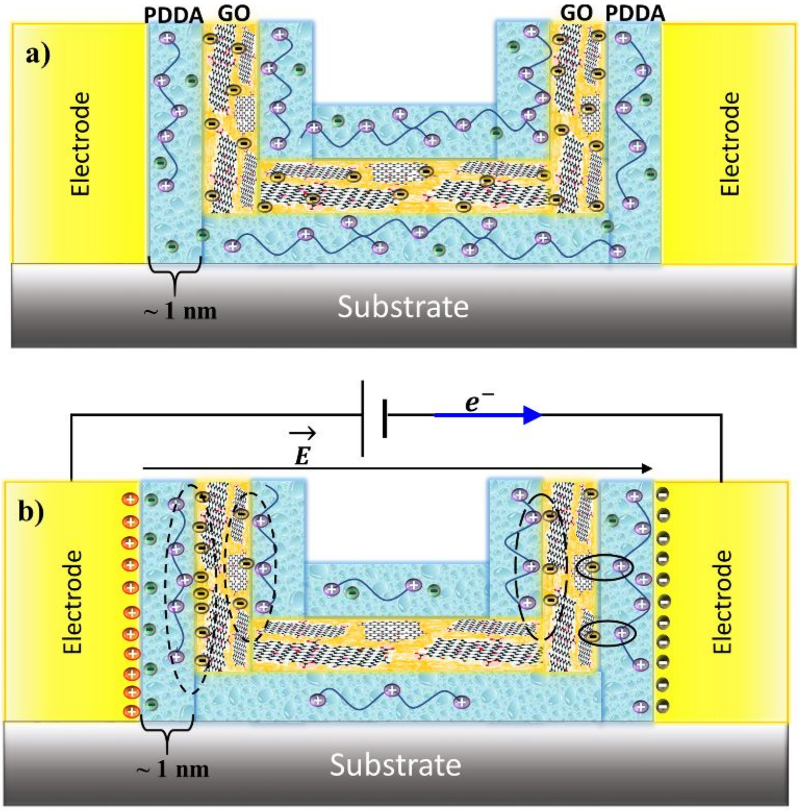

Figure S1. Illustration of the charge storage mechanism in the $(PDDA/GO)_n$ LbL structure: a) in the absence of applied potential difference; and b) with a potential difference applied between two electrodes.

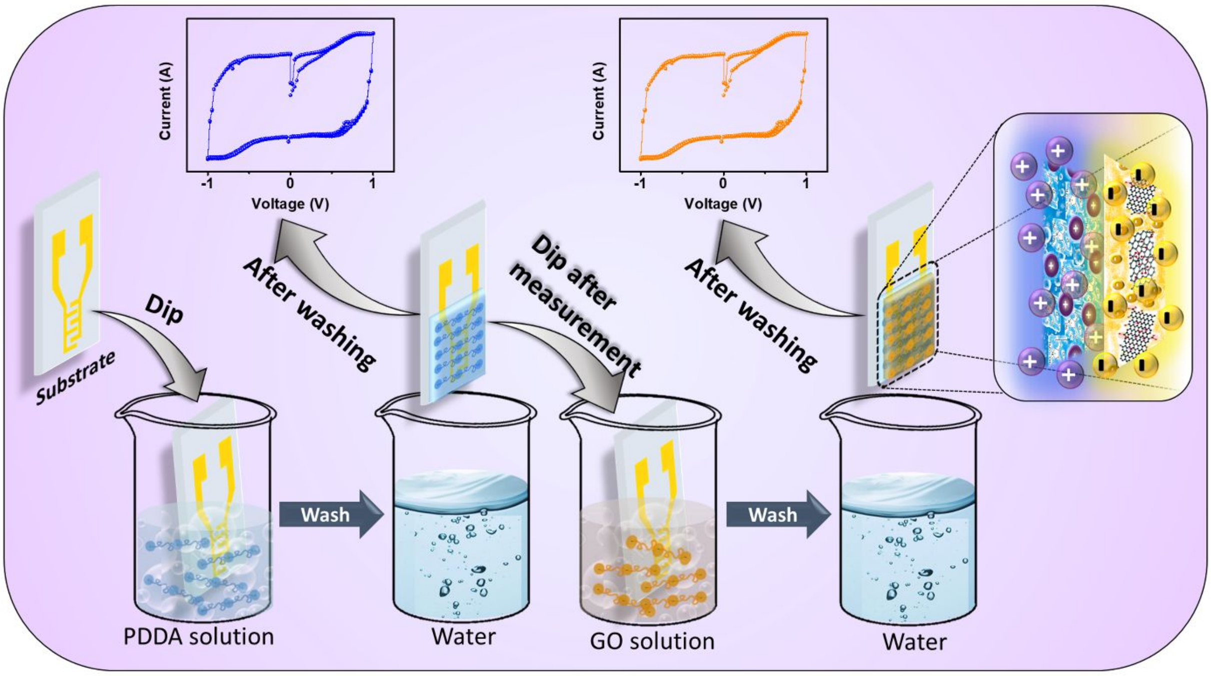

Figure S2. Schematic of the $(PDDA/GO)_n$ layer-by-layer assembly on IDEs. The multilayer formation was monitored by current–voltage (I–V) measurements taken at each deposition step.

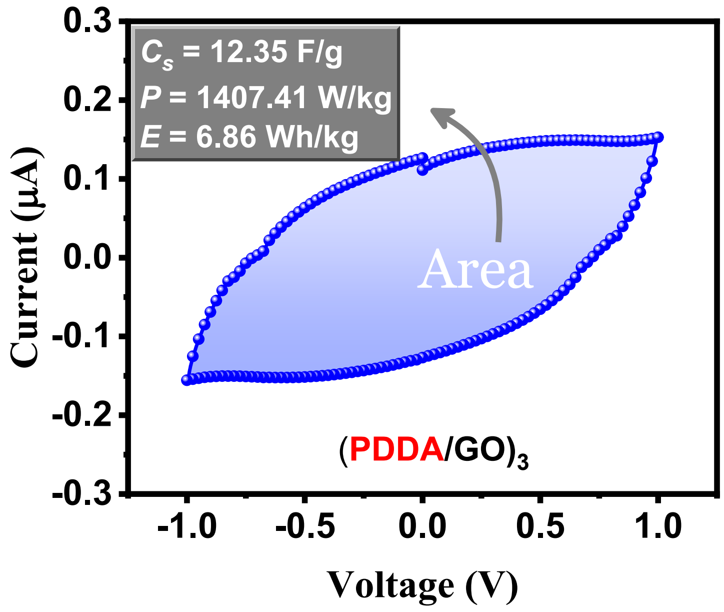

Figure S3. I–V curve of the $(PDDA/GO)_3$ hybrid solid-state supercapacitor, where PDDA is the outermost layer, measured at a scan rate of 55 mV·s⁻¹ within a voltage window of –1 V to +1 V. The specific capacitance (C□), power density (P), and energy density (E) values were determined as 12.35 F·g⁻¹, 1407.41 W·kg⁻¹, and 6.86 Wh·kg⁻¹, respectively, using Equations (1) – (4) in the main text.

Supplementary Notes on the Molecular Dynamics simulations

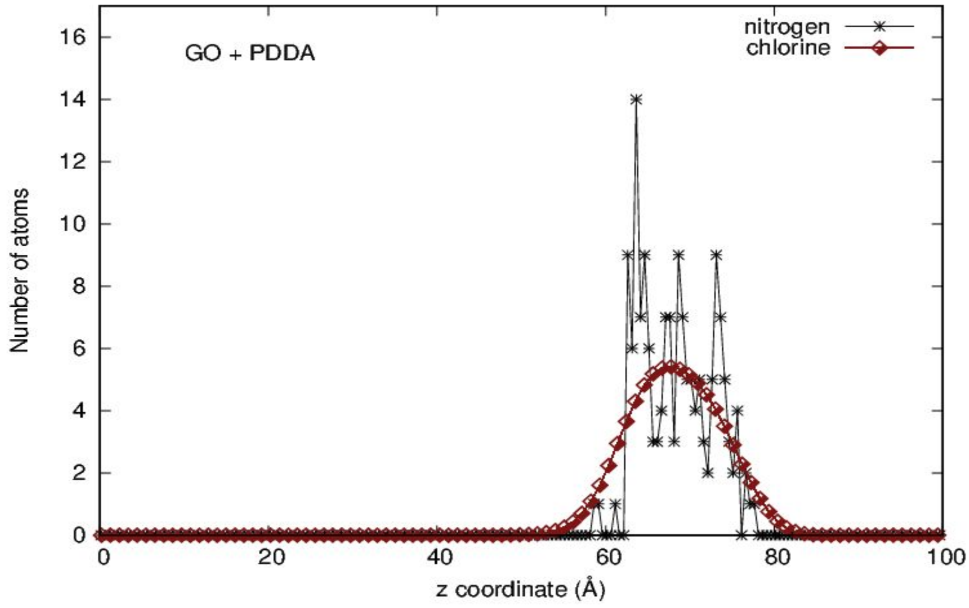

Figure S4. Distribution of nitrogen (black stars) and chlorine (brown diamond) atoms along the z direction for the PDDA/GO dry system, with chlorine atoms.

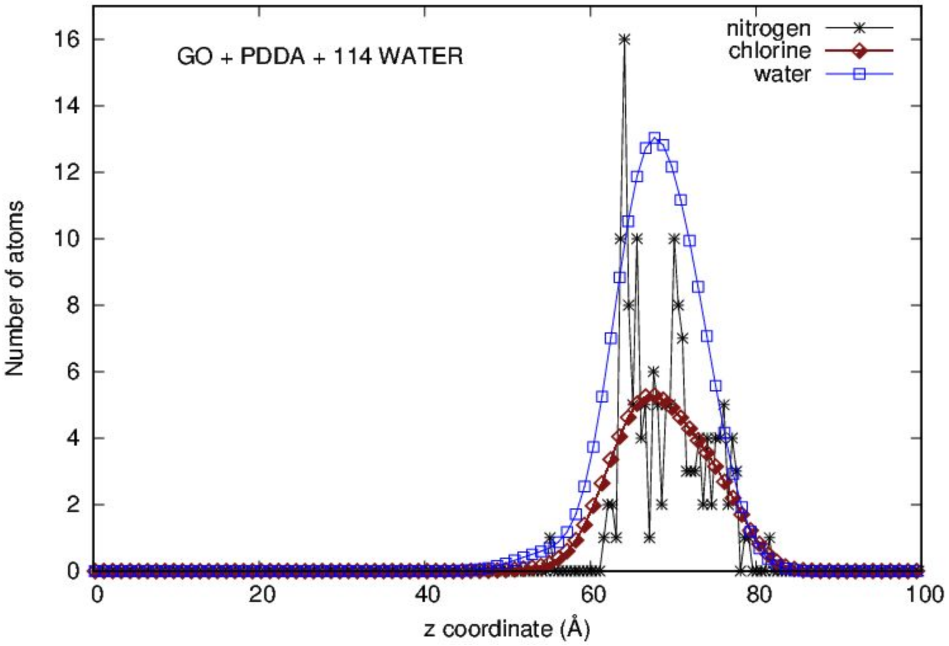

Figure S5. Distribution of nitrogen (black stars) atoms, chlorine (brown diamond) atoms and water molecules (blue squares) along the z direction for the PDDA/GO + 114 water molecules system, with chlorine atoms.

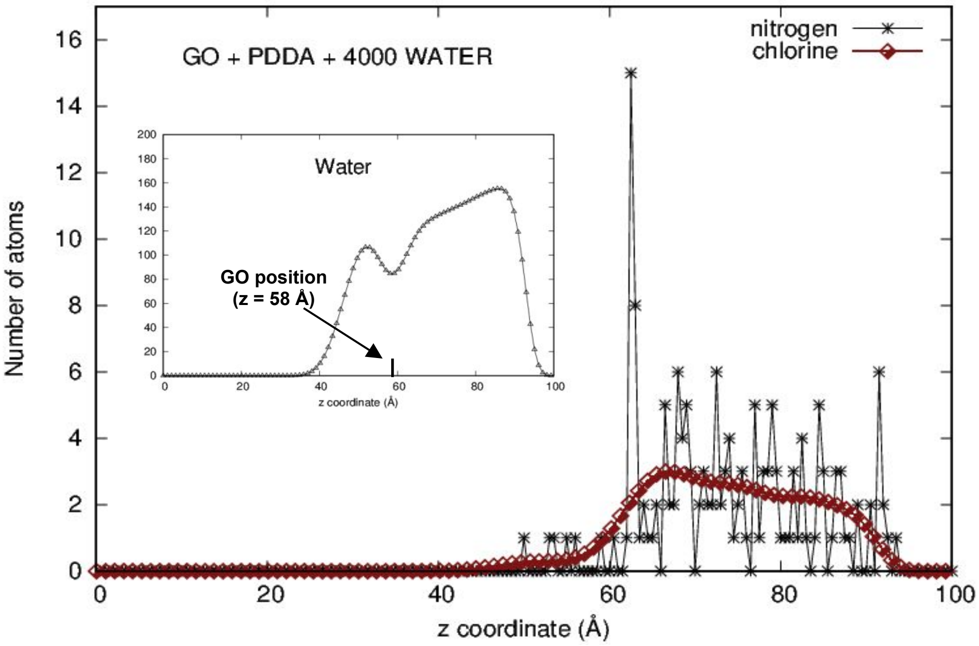

Figure S6. Distribution of nitrogen (black stars) and chlorine (brown diamond) atoms along the z direction for the PDDA/GO + 4000 water molecules system, with chlorine atoms. The inset shows the same distribution but for the water molecules.

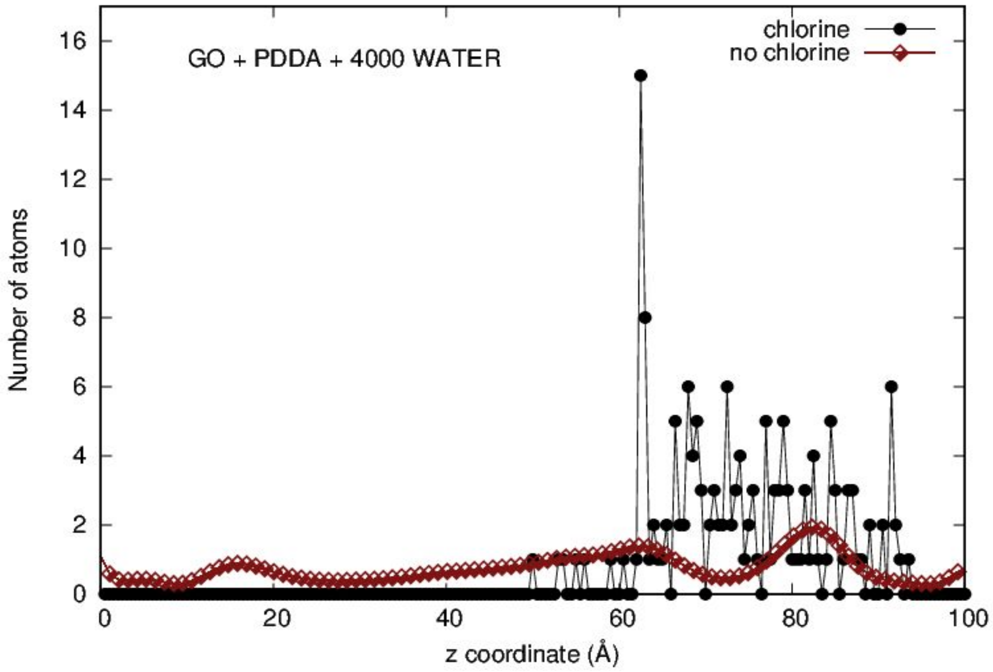

Figure S7. Distribution of nitrogen atoms along the z direction for the PDDA/GO + 4000 water molecules system, with (black circles) and without (brown diamonds) chlorine atoms.

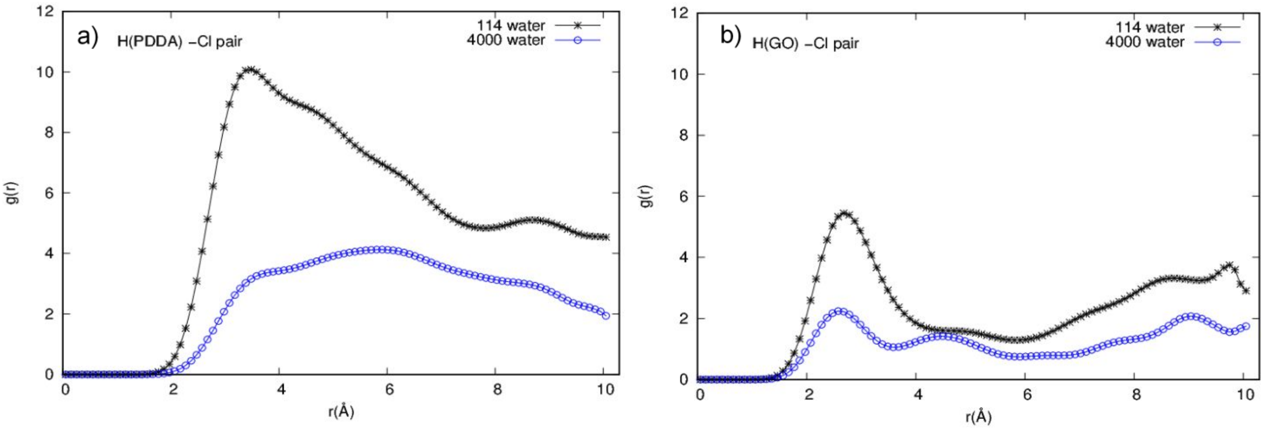

Figure S8. Radial distribution function, $g(r)$, for the hydrogens from PDDA and chlorine atoms H(PDDA)-Cl (a), and for hydrogens from hydroxyl groups of GO and chlorine atoms, H(GO)-Cl (b), considering 114 water molecules (black stars) and 4000 water molecules (blue circles).

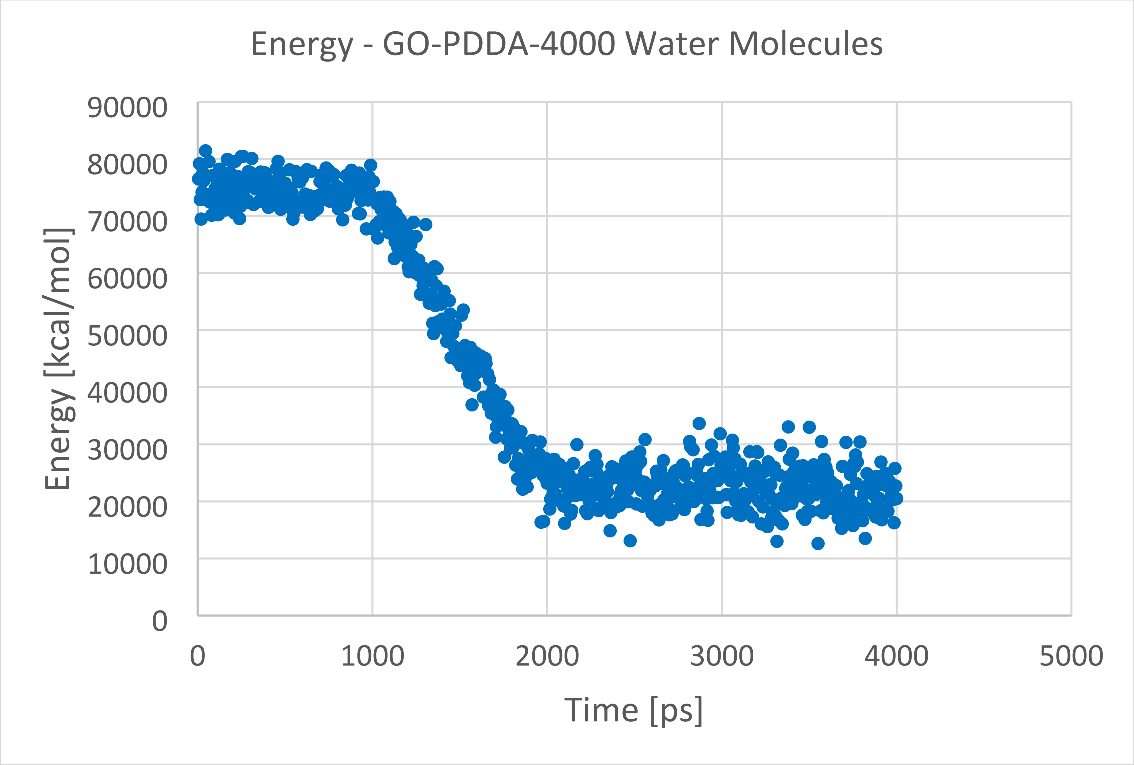

Figure S9. Curve of total energy versus time for the condition of GO-PDDA-4000 Water Molecules. As is evident, the total energy reaches a constant value within thermal fluctuations.

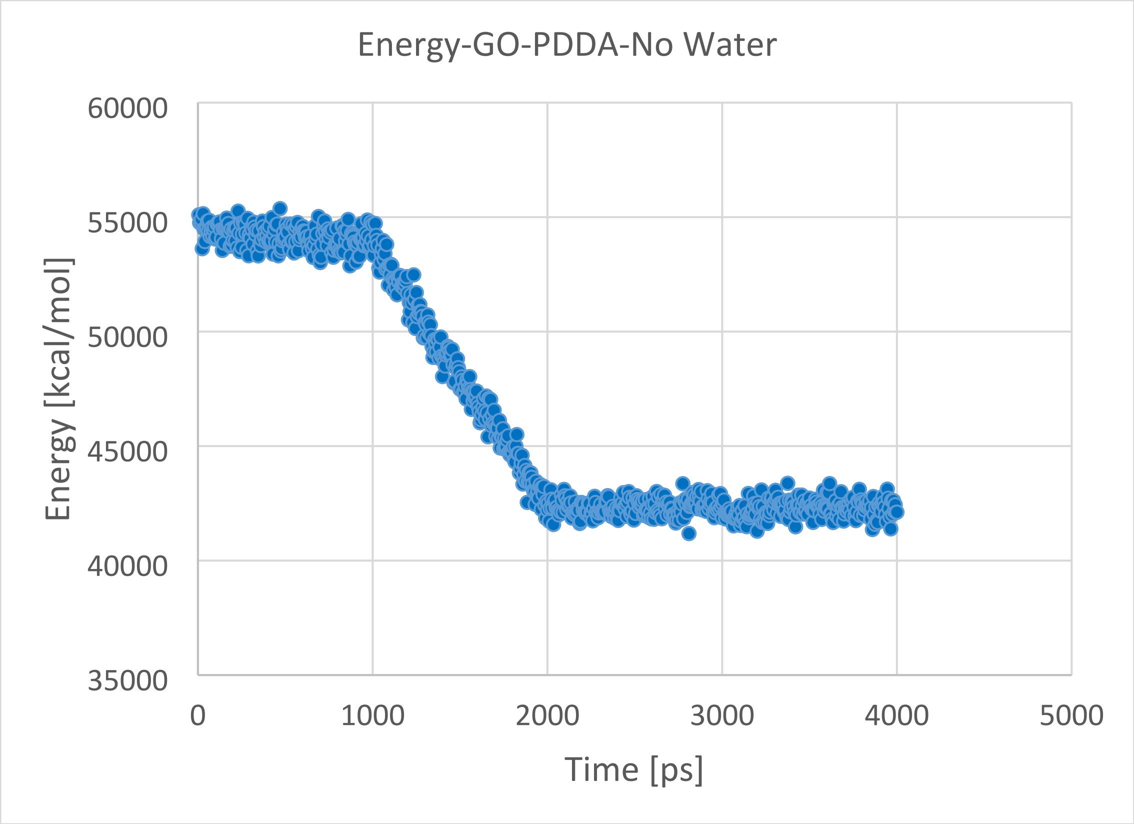

Figure S10. Curve of total energy versus time for the condition of GO-PDDA-No Water Molecules. As is evident, the total energy reaches a constant value within thermal fluctuations.
